# Recombinant Spider Silk: Promises and Bottlenecks

**DOI:** 10.3389/fbioe.2022.835637

**Published:** 2022-03-08

**Authors:** Maryam Ramezaniaghdam, Nadia D. Nahdi, Ralf Reski

**Affiliations:** ^1^ Plant Biotechnology, Faculty of Biology, University of Freiburg, Freiburg, Germany; ^2^ Cluster of Excellence livMatS at FIT – Freiburg Center for Interactive Materials and Bioinspired Technologies, University of Freiburg, Freiburg, Germany

**Keywords:** fibre, moss, recombinant production, expression systems, biomaterial, smart material, bioproduction

## Abstract

Spider silk threads have exceptional mechanical properties such as toughness, elasticity and low density, which reach maximum values compared to other fibre materials. They are superior even compared to Kevlar and steel. These extraordinary properties stem from long length and specific protein structures. Spider silk proteins can consist of more than 20,000 amino acids. Polypeptide stretches account for more than 90% of the whole protein, and these domains can be repeated more than a hundred times. Each repeat unit has a specific function resulting in the final properties of the silk. These properties make them attractive for innovative material development for medical or technical products as well as cosmetics. However, with livestock breeding of spiders it is not possible to reach high volumes of silk due to the cannibalistic behaviour of these animals. In order to obtain spider silk proteins (spidroins) on a large scale, recombinant production is attempted in various expression systems such as plants, bacteria, yeasts, insects, silkworms, mammalian cells and animals. For viable large-scale production, cost-effective and efficient production systems are needed. This review describes the different types of spider silk, their proteins and structures and discusses the production of these difficult-to-express proteins in different host organisms with an emphasis on plant systems.

## Introduction

Spider silks have fascinated scientists for decades due to their outstanding mechanical properties. A combination of high tensile strength and large extensibility makes them remarkably tough; they are five times stronger than steel and possess toughness threefold than that of Kevlar (Gosline et al., 1999; Vollrath and Knight, 2001). In addition to mechanical properties, spider silks have bio-properties, such as biocompatibility and slow degradability. They have been used as sutures for wound healing for centuries (Altman et al., 2003). Because of these properties, spider silks are regarded as a promising material for medical applications such as selective microbial-resistant coatings (Kumari et al., 2020), organic and degradable biosensors for biomonitoring of analytes in the body (Xu et al., 2019), wound healing (Öksüz et al., 2021), creating lenses useful for biological imaging (Tian et al., 2020), tissue engineering (Salehi et al., 2020) such as artificial blood vessels (Dastagir et al., 2020), nerve regeneration (Kornfeld et al., 2021; Millesi et al., 2021) and scaffolds creation (Gellynck et al., 2008). In addition, these silks have potential for use as smart materials. It was reported that they can be used in lithium-ion batteries to retain the capacity and decrease the volume expansion of silicon (Choi and Choy, 2020), for aerospace application (Mayank et al., 2021), as vision-based vibration sensors (Liu et al., 2020a), silk-based humidity sensors (Liu et al., 2020b), or protein-based adhesives for transparent substrates (Roberts et al., 2020). In addition, application of spider silk proteins in the cosmetic industry has been explored recently, for example as a new technology for face lifting (Qing et al., 2021).

### Spider Silks and Their Properties

**FIGURE 1:**
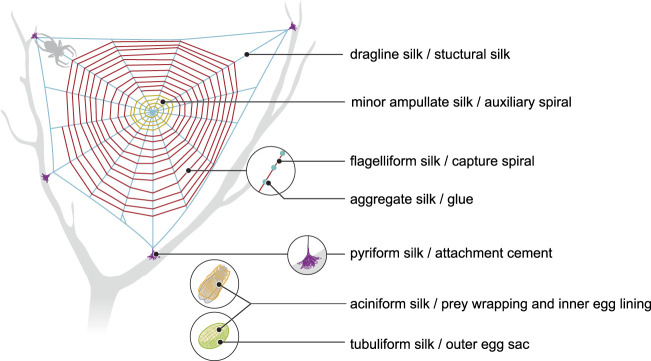
Schematic representation of different types of spider silk and their role in spider webs (compiled according to Vollrath and Knight 2001).

More than 41,000 species of spiders produce silks (Vierra et al., 2011). Seven different silks are produced (Figure 1), each of them having different properties. Each silk is produced by a specific gland and extruded from spinnerets located on the posterior end of the spider’s abdomen (Vollrath and Knight, 2001).

**Dragline silk** (major ampullate silk) is the most studied spider silk and makes the framework of the web (Figure 1). This silk has been at the center of attention for several years, since it is the strongest silk analyzed so far. Its estimated strength is 1,290 MPa (megapascal) in *Argiope trifasciata* (Hayashi et al., 2004). A variation is seen in the measured mechanical properties of silk fibres collected from nature (Table 1), which may be a result of the difficulties in collecting a particular type of silk for reliable mechanical testing. Another reason can be the regulation of spinning rate by the spider to make the property of the silk suitable for the specific task (Yarger et al., 2018). Moreover, it was suggested that various nutrition conditions (prey variation) may affect silk composition and mechanics. However, this assumption was rejected by Kono et al. (2019), who investigated mechanical properties after starvation or directly after feeding and found no effect of nutrition conditions on protein components and mechanical properties.

**TABLE 1:** Mechanical properties of seven types of spider silks.

Silk	Young´s modulus (GPa)	Strength (MPa)	Extensibility (%)	Toughness (MJ/m^3^)	References
Species of spider					
Dragline silk	—	906.9±	19.55 ± 5.02	84.28 ± 31.91	Kono et al. (2019)
*Araneus vetricosus*					
Dragline silk	8.3 ± 0.54	—	—	141.2 ± 0.77	Swanson et al. (2006a)
*Araneus diadematus*					
Dragline silk	9.3	1,290 ± 29	22	145	Hayashi et al. (2004)
*Argiope trifasciata*					
Dragline silk	3.4–11.5	1,030 ± 176	25–35	149 ± 25	Kerr et al. (2018)
*Nephila pilipes*					
Dragline silk	—	1,030 ± 206	—	73.22 ± 7.60	Kerr et al. (2018)
*Nephila plumipes*					
Dragline silk	13.8 ± 3.6	1,215 ± 233	—	11.2 ± 30	Swanson et al. (2006b)
*Naphila clavipes*					
Dragline silk	10.2 ± 0.75	—	—	180.9 ± 11.19	Swanson et al. (2006a)
*Latrodectus hesperus*					
Minor ampullate silk *Argiope trifasciata*	8.5	342	54	148	Hayashi et al. (2004)
Minor ampullate silk *Latrodectus hesperus*	3.9 ± 2.9	245.4 ± 120.5	0.57 ± 0.02	66.7 ± 46.4	Vienneau-Hathaway et al. (2017)
Minor ampullate silk	2.6 ± 1.3	174.4 ± 17.6	0.54 ± 0.02	43.6 ± 20.1	Vienneau-Hathaway et al. (2017)
*Latrodectus geometricus*					
Minor ampullate silk *steatoda grossa*	2.1 ± 0.7	251.3 ± 106.2	0.74 ± 0.02	57.3 ± 20.7	Vienneau-Hathaway et al. (2017)
Flageliform	0.003	500	270	150	Gosline et al. (1999)
*Araneus diadematus*					
Flagelliform	0.012–0.08	800 ± 100	≥200	—	Perea et al. (2013)
*Argiope trifasciata*					
Pyriform	0.2 ± 0.1	100 ± 40	50–80	61 ± 47	Greco et al. (2020)
*Cupiennius salei*					
Aciniform	9.6	687	86	367	Hayashi et al. (2004)
*Argiope trifasciata*					
Clyndriform	9.1	390	40	128	Zhao et al. (2006)
*Argiope bruennichi*					
Cylindriform	8.7 ± 0.9	400 ± 50	5–20	—	Nimmen et al. (2005)
*Araneus diadematus*					

GPa, gigapascal; MPa, megapascal; MJ, megajoule; m^3^, cubic meter.

**FIGURE 2:**
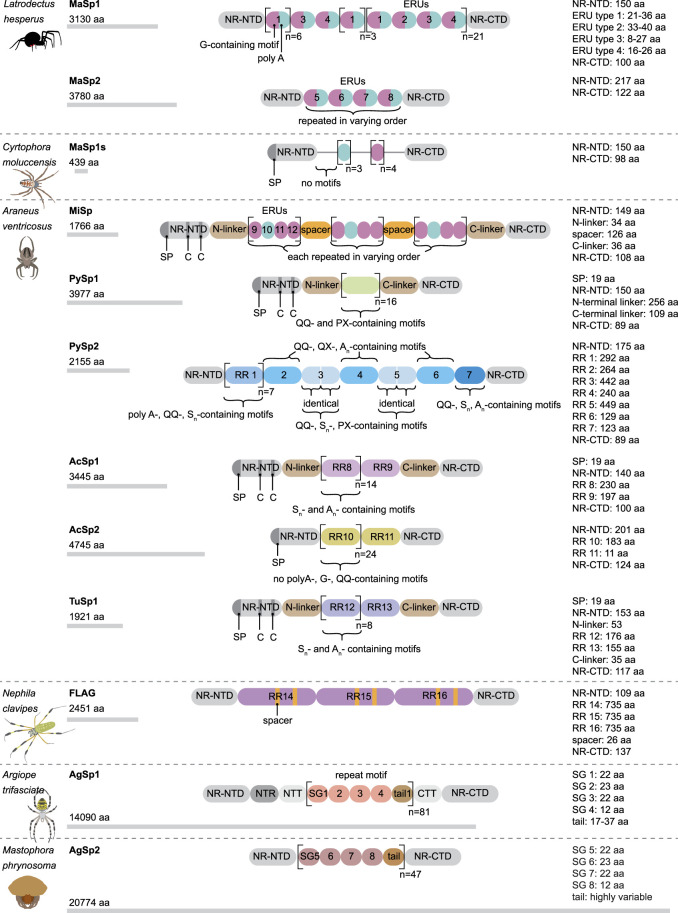
Schematic modular structure of different spidroins. aa, amino acid; G, glycine; C, cysteine; NR-NTD, non-repetitive N-terminal domain; NR-CTD, non repetitive C-terminal domain; SP, signal peptide; RR, repetitive region; ERU, ensemble repeat unit; SG, subgroup.

This silk is composed of two proteins, major ampullate spidroin 1 (MaSp1) and major ampullate spidroin 2 (MaSp2), with estimated molecular masses of over 250 kDa (Table 2). MaSp1 and MaSp2 proteins in *Latrodectus hesperus* consist of a non-repetitive N-terminal domain (NR-NTD), a large and highly repetitive core region and a non-repetitive C-terminal domain (NR-CTD) (Ayoub et al., 2007) (Figure 2). The N-terminal region contains non-repetitive amino acids and may play a crucial role in the transport of the spidroin into the glandular lumen (Ayoub et al., 2007; Zhang et al., 2013). N-terminal domains are formed as antiparallel dimers due to surface charges and control protein interaction and elongation (Hagn et al., 2011). Dimerization of the N-terminal domain is dependent on pH. This region responds to ionic and mechanical changes and can promote the solubility of spidroins at neutral pH (Askarieh et al., 2010; Bauer and Scheibel., 2017; Chakraborty et al., 2020). Three conserved residues of the N-terminal domain of dragline silk in *Euprosthenops australis*, Glu79, Glu84 and Glu119, are protonated to form a homodimer from a monomer when the pH changes from 7 to lower pH values (Jiang et al., 2019). N- and C-terminal domains lead to an increase in Young’s modulus, stress, and toughness of recombinant proteins (Zhu et al., 2020). Sequence alignments of N- and C-terminal domains of spidroins show that these domains are highly conserved (Ayoub et al., 2007). The core repetitive region in MaSp1 is composed of four types of ensemble repeat units (ERUs) which are tandemly arrayed in a consistent pattern. This pattern is iterated 20 times with near-perfect fidelity. Each ensemble consists of a glycine-rich region followed by a poly-A region. Amino acid motifs of each ensemble are GGX (X = A, Q, or Y), GX (X = Q, A, or R) and poly-A. Similar to MaSp1, the core repetitive region of MaSp2 is organized into four types of ERUs, which are more variable than those of MaSp1. Moreover, they are not always tandemly arrayed in the same order. Core region motifs of MaSp2 comprise GPX (X = G or S), GGX (X is usually A), GSG, QQ and poly-A (Ayoub et al., 2007). Techniques such as X-ray diffraction, NMR measurements and transmission electron microscopy (TEM) revealed aligned nanocrystalline β-sheets in the predominant crystalline component (Van Beek et al., 2002; Trancik et al., 2006). Further studies showed that mechanical properties of spider dragline silk correlate with their molecular structure (Yarger et al., 2018; Htut et al., 2021). Poly-A motifs form β-sheet structures (Trancik et al., 2006; Van Beek et al., 2002), in which large numbers of hydrogen bonds are created between the backbone amine and carbonyl groups (Zhang et al., 2014). The strength of dragline silk directly correlates with this β-sheet structure (Yarger et al., 2018). On the contrary, GGX and GPX peptide motifs, which are less orientated and amorphous, have α helical and type II β-turns structures, respectively. These are not as constrained as β-sheet structures (with high density of hydrogen bonds) so that they grant extensibility to dragline fibres (Jenkins et al., 2010; Gray et al., 2016; Yarger et al., 2018) (Table 3). Cysteine residues in the C-terminal domain are involved in intermolecular disulfide formation. The pH value, salt concentration, and shear-force-induced partial unfolding of the disulfide-bridged dimeric C-terminal domain control the correct alignment of polyA/polyGA sequences to form microcrystalline structures facilitating the assembly of fibres (Hagn et al., 2011). The properties of this silk can change upon exposure to water (vapour or liquid), leading to an increase in diameter and a decrease in length. This behaviour is called supercontraction, during which a loss of molecular structural order is induced and fibre stiffness is decreased. It is proposed that this property is related to the content of proline, making type II β-turns structures (Liu et al., 2005; Blackledge et al., 2009; Lang et al., 2017).

**TABLE 2:** Different types of spider silk proteins and their properties.

Silk type	Role of silk in web	Protein	Molecular mass	Number of amino acid	Spider species	Motifs	References
Dragline	Framework of the web	MaSp1	>250	3130	*Latrodectus hesperus*	-GGX (X = A, Q, or Y)	Ayoub et al. (2007)
						-GX (X = Q, A, or R)	
						-poly-A	
Dragline	Framework of the web	MaSp1s	40	439	*Cyrtophora moluccensis*	-GGX (X = A, Q, or Y)	Han et al. (2013)
						-GX (X = Q, A, or R)	
						-poly-A	
Dragline	Framework of the web	MaSp2	>250	3780	*Latrodectus hesperus*	-GPX (X = G or S)	Ayoub et al. (2007)
						-QQ	
						-GGX (X is usually A)	
						-GSG	
						-poly-A	
Minor ampullate (MI)	Auxiliary spiral to stabilize the scaffold	MI	>250	1766	*Araneus ventricosus*	-GGX	Chen et al. (2012)
						-GGGX	
						-GX	
						-PolyA	
						-spacer	
Flagelliform	Capture spiral of the orb web	Flag	>250	2451	*Nephila clavipes*	-GPGGX	dos Santos-Pinto et al. (2018); Hayashi and Lewis (1998); Hayashi and Lewis (2000)
						-GGX	
						-Spacer motif	
Pyriform	Attachment of silk fibres to surface	PySp1	400	3977	*Araneus ventricosus*	-QQ containing motif	Wang et al. (2019)
						-Proline-rich regions	
						-N-linker	
Pyriform	Attachment of silk fibres to surfaces	PySp2	212	2155	*Araneus ventricosus*	-QQ containing motif	Wang et al. (2020)
						-Proline-rich regions	
Aciniform	Prey wrapping and inner silk of egg sac	AcSp1	330	3445	*Araneus ventricosus*	Motifs are long and complex. poly-A, GGX, GPX and poly-GA are not present	Wen et al. (2018)
Aciniform	Prey wrapping and inner silk of egg sac	AcSp2	476	4746	*Araneus ventricosus*	Motifs are long and complex. poly-A, GGX, GPX and poly-GA are not present	Wen et al. (2020)
Tubuliform	Egg sac	TuSp1	180	1921	*Araneus ventricosus*	-A_n_, S_n,_ SA_n_, AX, (SG)_n_	Wen et al. (2017)
						-linker	
Aggregate	Glue of capture spiral	AgSp1	450–1,400	14090	*Argiope trifasciata*	-GPXG at the beginning of subgroups	Stellwagen and Renberg (2019)
						-The tail regions contain GGQ, PGG, GPG and QGP motifs	
						-QQ motifs	
Aggregate	Glue of capture spiral	AgSp2	—	20774	*Mastophora phrynosoma*	-	Stellwagen and Burns (2021)

**TABLE 3:** Different motifs in spider silk proteins and their roles in spider silk.

Motif	Secondary structure	Structural role of motif	Mechanical properties	References
Poly-A	β-sheet 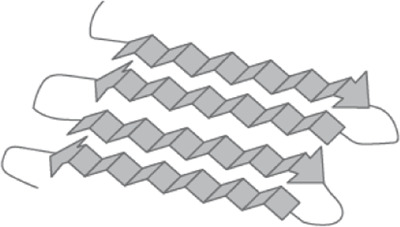	Crystalline 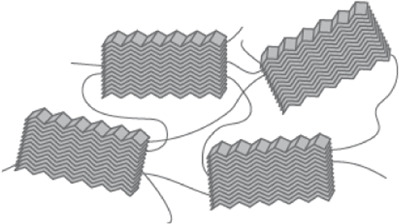	Tensile strength	Van Beek et al. (2002); Yarger et al. (2018)
GGX GX	α-helix 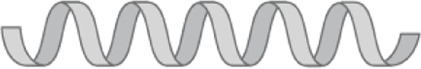	Amorphous 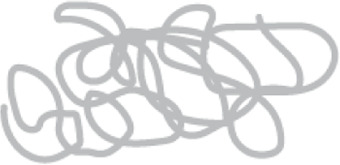	Elasticity	Gray et al. (2016); Yarger et al. (2018)
GPX GPGGX	Type II β-turns 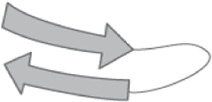	Elastic 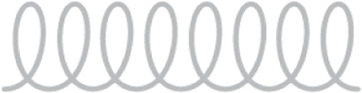	Elasticity, toughness and supercontraction	Hayashi and Lewis (1998); Ohgo et al. (2006); Jenkins et al. (2010)
QQ-containing motif	α-helix or β-sheet	—	Self-aggregation of proteins into fibres	Wang et al. (2019)
(PX)_n_	Random coil	—	—	Wang et al. (2019); Zhu et al. (2020)
Spacer	α-helix	—	Helps to form fibres or contribute to the strength of fibres	Vienneau-Hathaway et al. (2017)

A short type of dragline silk protein called MaSp1s, consisting of 439 aa and a molecular mass of 40 kDa, has been identified in *Cyrtophora moluccensis* (Table 2). It contains a non-repetitive N-terminal domain (149 aa), core region (192 aa) and a C-terminal domain (98 aa) (Figure 2). Two terminals are homologous to that of other dragline spidroins. There is an apparent signal peptide in the N-terminal region of MaSp1s and a putative cleavage site between amino acids 24 and 25. An obvious repetitive region is not seen in this protein. It has only 7 short repeat units which are not extremely homogenized. However, this protein comprises all the motifs of MaSp1 such as GGX (X = A, Q, or Y), GX (X = Q, A, or R) and polyA. MaSp1s has less repetitive units as other dragline spidroins, so that they may not have a key role in determining the mechanical properties of the silk. The abundance of this small protein is lower than that of MaSp1 and MaSp2, implying that it may not have a dominant effect on the strength and elasticity of the silk (Han et al., 2013).

With presenting the first genomes in the genus *Araneus*, the full spidroin gene set was obtained and confirmed with the transcriptome of silk glands as well as the proteome of the silk (Kono et al., 2021). As a result, the full length (7854 bp) of a new paralog in the *MaSp* gene family, *MaSp3,* was isolated. This gene is found in *Araneus ventricosus* and *Argiope argentata* (Kono et al., 2019) and was previously reported partially (Collin et al., 2018). *MaSp3* is highly expressed in the major ampullate gland and the MaSp3 protein is the most abundant in dragline silk. A Principal Component Analysis (PCA) revealed that MaSp3 does not have a direct contribution to the mechanical properties of this silk. Only a limited homology is seen between terminal domains of MaSp3 and MaSp1 and MaSp2 in *A. vetricosus* (Kono et al., 2019). The existence of some additional proteins was confirmed within dragline silk, such as alpha-2-macroglobulin 2 and peroxidasin, which are found in the peripheral layer and the core region of the caddisfly silk fibre (Kono et al., 2019). Peroxidasin contributes to the silk post-draw dityrosine crosslinking (Wang et al., 2014).

With a multiomics approach, genomes of four closely related Nephilinae, gland transcriptomes, and proteomes of dragline silk are available (Kono et al., 2021), which will contribute to future research. With this high-quality genome analyses, a conserved MaSp3B was reported in the genera *Trichonephila* and *Nephila*. The important residues, such as Aspartic acid40, Lysine65, Glu79, and Glu119, which have impact on the N-terminal domain dimerization, are conserved in this protein (Kono et al., 2021). Non-canonical silk constituents termed SpiCEs were found in the spider silk. Four SpiCEs proteins are highly expressed exclusively in the MA gland. They are not homologuous to described spidroins (Kono et al., 2019). A new SpiCE-NMa1 (SpiCE Nephilinae Major Ampullate) was reported in Nephilinae spiders, which are nonhomologous to those of *A. ventricosus.* Composite films of recombinant MaSp proteins and SpiCE-NMa1 were produced to investigate the role of these new low-molecular-weight components. It was reported that these elements can increase the tensile strength of the composite film 2-fold. SpiCE contributes to the interaction between repeat regions, preferentially in the amorphous region rather than in crystalline structure. These intermolecular interactions lead to a decrease in the molecular weight between crosslinking points, and thus the strength and modulus of the artificial film are enhanced (Kono et al., 2021). New elements found in spider silk can confirm it as a multicomponent material, which is more complex than expected previously (Kono et al., 2019; Kono et al., 2021).

**Minor ampullate silk (MI)** is used by spiders to make the auxiliary spiral of the orb-web to stabilize the scaffold (Figure 1). MI silk plays a similar role in the spider web as MA silk but does not achieve its high biophysical properties (Chen et al., 2012). The analysis of the *MiSp* gene shows the presence of an unusually large intron of 5628 bp. Generally, intron length and expression level are negatively correlated (Castillo-Davis et al., 2002; Urrutia and Hurst, 2003; Marais et al., 2005). However, *MiSp* genes are likely highly expressed. Proteins of this silk (MiSp) have molecular masses of over 250 kDa (Table 2). Minor ampullate protein sequences in *A. ventricosus* comprise a non-repetitive N-terminal domain, one N-linker, three repetitive regions, two non-repetitive spacer regions, one C-linker and a C-terminal domain (Chen et al., 2012) (Figure 2). The N-terminal region of MiSp in solution at pH 7.2 contains five helices. Cys25 and Cys96 form an intramolecular disulfide bridge. Key residues for pH-dependent dimerization in the N-terminal domain of MiSp were considered as Glu76, Glu115, and Glu73, which are different from those of MaSp1. However, the monomer-to-dimer conversion has the same mechanism in MaSp and MiSp spidroins. An anti-parallel homodimer structure is seen in the five-helix of this region at pH 5.5 (Otikovs et al., 2015). There is no cysteine in the C-terminal domain of *A. ventricosus* MiSp. The C-terminal area can dimerize via hydrophobic interactions. MiSp has three repetitive regions, which are interrupted by two non-repetitive spacer regions. Each repetitive region can be categorized into four types of ERUs [GGX-GGX-GX, (GX)_n_ oligoA-(GX)_n_, GGX-GGX-GGGX and (GX)_n_] iterating in a non-regular manner. Reoccurring overall patterns are seen in the repetitive region. However, this protein, in comparison with *L. hesperus* MaSp1, lacks higher order organization. Repetitive regions are dominated by polyA, GGX, GGGX and GX motifs. There is no proline in this structure, which is why this silk does not supercontract in water. There are two spacer regions having 100% identity even at the nucleotide level. These spacers have no identity to any proteins except other spacer regions in the proteins of MiSp or Flag. The spacer region is not repetitive but only has a single tandem repeat (AAASS). Spacer regions are predicted to contain α-helices (Chen et al., 2012). The roles of these regions are not well characterized but it has been hypothesized that they help to form fibres (Vienneau-Hathaway et al., 2017) (Table 3).

**Flagelliform silk** has the highest extensibility among all silks produced by orb-weaver spiders and is used as the capture spiral of the web (Figure 1). This silk is not as strong as dragline silk, but it is multiple times more extensible. This fibre can be stretched to 250% (Gosline et al., 1999; Rising and Johansson, 2015) (Table 1) and dissipates the impact energy of prey. As an example, a honey bee with flight velocity of 3.1 m/s and 120 mg body weight crashes into the web with a 0.55 mJ kinetic energy. Flag silk (with 1–5 µm diameter) can withstand that massive impact. This outstanding resilience helps the spider to catch a prey even bigger than itself (Römer and Scheibel, 2008). Therefore, this silk could have an application for dampening vibration in material development (Hauptmann et al., 2013b).

The *Flag* gene is one of the longest spidroin genes, it comprises a total exonic region of 22.5 kb in the *A. ventricosus* genome (Kono et al., 2019). The corresponding protein of this species has not been characterized yet. In contrast, with mass spectrometry, the sequence and domains of the Flag protein of *Nephila clavipes* were identified. It contains an N-terminal domain, three modules of repetitive regions, two spacers in each repetitive region and a C-terminal domain (Figure 2). N-terminal and C-terminal regions have three α-helices and one small helical section, respectively (dos Santos-Pinto et al., 2018). Motifs of repetitive regions are GPGGX and GGX. GPGGX likely forms type II β-turns, which may form β spirals. It is hypothesized that this spring-like helix is the basis for the elasticity of spider Flag silk (Hayashi and Lewis, 1998) (Table 3). Some studies show amorphous shapes without crystalline structure in the repetitive regions (Römer and Scheibel, 2008). It is suggested that abundance of proline residues prevents forming crystalline β-sheet (Ohgo et al., 2006). However, the presence of polyglycine II nanocrystals was demonstrated in the flagelliform silk of *A. trifasciata* (Perea et al., 2013). This silk does not contain a polyA motif providing the strength of dragline silk. Each repetitive region contains two spacers with 100% similarity in their amino acid composition. Spacers have charged amino acids which are assumed to contribute to the strength of fibres by crosslinks between Flag proteins (Hayashi and Lewis, 1998; Adrianos et al., 2013; dos Santos-Pinto et al., 2018).

Previously, gene and mRNA length of *Flag* in *N. clavipes* were estimated at 30 and 15.5 kb, respectively. The *flag* gene has 13 exons. The corresponding protein was reported to have three motifs: GPGG(X)_n,_ GGX and 28 spacers. Iterations of these three motifs are organized into complex ensemble repeats. Each ensemble repeat is encoded by a single exon. The exons have similar lengths (∼1,320 bp) (Hayashi and Lewis, 1998; Hayashi and Lewis, 2000).

The first genome of *N. clavipes* was sequenced to investigate spidroin genes. The authors catalogued a collection of 28 spidroins and new repetitive elements. This collection of data was complemented by expression profiling of the silk gland and shows a diversity of spidroin genes (Kono et al., 2021). A new flagelliform gene called *FLAG-b* was found in the *N. clavipes* genome. Transcripts of *FLAG-b* are highly abundant in venom glands. Previously, two spidroin-like proteins, SmSp1 and SmSp2b, were found in the venom gland of the velvet spider. These findings suggest that FLAG-b can be a new type of venom gland-expressed spidroin (VeSp) evolving roles beyond silk-related functions. Proteomic studies are needed to investigate whether this protein is in the venom gland. Characterization and functional identification of this protein may open a new venue for using spidroins in human medical applications (Babb et al., 2017).

**Pyriform silk** forms attachment disks used as cement by spiders (Figure 1). The components of this silk are dry fibres and wet glue, both produced by the same gland. The glue dries immediately and forms a hardened disc. This glue silk is used by spiders to join different fibres or attach dragline to surfaces (Geurts et al., 2010; Wolff et al., 2015). This silk consists of two proteins, PySp1 and PySp2. PySp1 has a molecular mass of 400 kDa. In *A. ventricosus* it comprises five regions, a non-repetitive N-terminal domain, a long N-terminal linker, a central largely repetitive region, a short C-terminal linker, and a non-repetitive C-terminal domain (Figure 2). The presence of a signal peptide cleavage site in the N-terminal region was predicted. All silk proteins need to go through the ER and the secretory pathway and the signal peptide paves this way. In the N-terminal region 5 α-helices were predicted. In contrast, the C-terminal region contains 4 α-helices. Two N-terminal cysteines were detected between helix 1 and 2, and in helix 4. PySp1 exhibits two linkers, a long N-linker and a short C-linker. The N-terminal linker consists of two types of repeats, QQQYEXSQASIA and QQQYXXSQQQASIX. This linker contains 5 α-helices and is hypothesized to control the protein to form fibre or glue through self-assembly. The core repetitive region of PySp1 spidroin is made up of sixteen remarkably homogeneous units. Two motifs are seen in this region, proline-rich motifs (PXPXP) and QQ-containing motifs. The (PX)_n_ motifs seem to form random coils and QQ-containing regions form α-helix or β-sheet conformations. Glutamin segments seem to have a role in spidroin self-assembly (Wang et al., 2019; Zhu et al., 2020) (Table 2 and 3).

The *A. ventricosus* PySp2 has a molecular mass of 212 kDa and lacks the long linker regions. PySp2 has a more complex core repetitive region than PySp1. This protein has seven repetitive regions which can be classified into four types: The repetitive regions 2, 4, and 6 have the same repeats (containing QQ, QX and A_n_). The repetitive regions 3 and 5 are also similar in repeat sequences (Figure 2). The repetitive region 7 is the shortest one lacking glutamine and may perform specific functions. The three regions 2, 4, and 6 may contribute to PySp2 aggregation or self assembly due their QQ-containing regions (Wang et al., 2020).

**Aciniform silk** acts as prey wrapping and forms the inner silk of the egg sac (Figure 1). This silk is one of the toughest silks, 367 MJ/m^3^ (megajoule per cubic meter), among seven silks (Table 1). Aciniform silk protein (AcSp1) has a calculated mass of ∼330 kDa with 3445 aa (Table 2). Similar to Pyriform, this protein is composed of five regions, a non-repetitive N-terminal domain, a N-terminal linker, a central largely repetitive region, a short C-terminal linker, and a non-repetitive C-terminal domain (Figure 2). The N-terminal region has a signal peptide with 23 aa. The analyses show five α-helices and two cysteines in the locations corresponding to helix 1 and 4. The non-repetitive C-terminal domain has four α-helices. Two terminals of AcSp1 from *A. ventricosus* are homologous to that of other spidroins and species. The repetitive region contains 15 iterated repeats units. The first 14 repeats have the same length (230 aa) and the last one is 197 aa long. There are five α-helices in each repeat. Repeat units are highly conserved, and several repeats are 100% identical to each other. In the alignment of 15 repeats, only 59 sites are variable. Most of the variation belongs to the first repeat unit. Amino acid motifs such as poly-A, GGX, GPX and poly-GA, which are abundant in dragline, Flag and MiSp proteins, are not present in the AcSp1 protein (Wen et al., 2018).

Recently, a second type of protein (AcSp2) was identified in *A. ventricosus* with a molecular mass of 476 kDa. This protein has 4746 aa composed of three regions: an N-terminal region with a predicted signal peptide, a core repetitive region comprising 25 repeat units with extreme intragenic homogenization and a C-terminal region (Wen et al., 2020).

**Tubuliform** (**cylindriform**) **silk** forms the outer shell of the egg case (Figure 1). This is the only silk produced during a specific period of the female spider’s life, the reproductive season (Tian and Lewis, 2006). This silk is robust and able to protect offspring from predators, temperature fluctuation and parasitoid invasion. Tubuliform silk protein (TuSp1) is predicted to have a molecular mass of 180 kDa (Table 2). TuSp1 in *A. ventricosus*, like PySp1, has five regions, a non-repetitive N-terminal domain, a short N-terminal linker, a central repetitive region, a short C-terminal linker, and a non-repetitive C-terminal domain (Figure 2). The N-terminal domain has five α-helices, while there are five α-helices and three β strands in the C-terminal region (Wen et al., 2017). In the N-terminal domain two conserved cystein residues were identified in helix 1 and helix 4. The core region of this protein is dominated by nine tandem repeats. These repeats are highly conserved, >90% identical at amino acid level. Typical motifs such as polyA, GGX, GX and QQ, which were identified in other spidroins, are not seen in the TuSp spidroins. Instead, common sequence motifs in the TuSp repetitive sequence are A_n_, S_n_, SA_n_, AX and (SQ). Pyriform, aciniform and tubuliform spidroins have long and complex repeats. Analysis of repeat regions across species demonstrates extreme homogeneity of intragenic repeats in the proteins of these three silks (Ayoub et al., 2013; Wen et al., 2017).

**Aggregate silk** is a kind of glue to aid in prey capture (Figure 1). This glue comprises two proteins, AgSp1 and AgSp2, which are modified members of the spidroin family but they are not spun into fibres (Collin et al., 2016). The predicted mass is 450–1,400 kDa (Tillinghast et al., 1992) (Table 2). In *A. trifasciata*, AgSp1 has 14,090 amino acids and consists of an N-terminal region, N-terminal repeats (NRP), an N-terminal transition (NTT, a region with degenerate, repeat-similar structure), repetitive regions, a C-terminal transition (CTT) and a C-terminal region (Figure 2). N- and C-terminal regions are conserved across spidroins. Following the N-terminal region, there is a short region (586 aa) including repetitions of TGSYITGESGSYD. In the repetitive region two similar distinct motifs were predicted. This region includes 43 iterations of repeat motif 1 (129 aa) and 38 iterations of repeat motif 2 (113 aa). Repeat motifs contain four subgroups (SGs) and a variable tail region. Each subgroup begins with GPXG. The tail region contains GGQ, PGG, GPG and QGP motifs and poly-threonine stretches on both ends. The C-terminal transition region has the same organization as the internal repeats (Stellwagen and Renberg, 2019).

*Mastophora phrynosoma*, also called bolas spider, uses a fascinating hunting technique to capture moths. Females apply a single and large droplet of glue, which is suspended at the end of the silk thread. The bolas spider AgSp2 is the longest spidroin discovered so far. A massive intron of 31.5 kb was predicted within the gene. AgSp2 spidroin from this orb weaver spider has 20,774 amino acids (encoded by nearly 62 kb of genomic DNA). It remains a mystery what functions this long length has. This protein is comprised of ∼47 repeats and does not have glutamine-rich regions seen in the other reported AgSp2 of classic orb weaving species (Stellwagen and Burns, 2021). Glutamine was hypothesized to promote self-aggregation of spidroins into fibres (Geurts et al., 2010). Previously, two short aggregate proteins were reported with 407 and 715 aa in *Nephila clavipes* (Choresh et al., 2009).

### Post-Translational Modifications of Spider Silk Protein

Post-translational modifications (PTMs) are covalent modifications that change the properties of proteins. This chemical event ranges from enzymatic cleavage to adding a chemical group such as glycosyl, phosphoryl, acetyl or methyl. PTMs have important roles in the structure and function of proteins (Ramazi and Zahiri, 2021). By Concanavalin A, it was found that the core and shell of dragline silk in *N. clavipes* is glycosylated (Sponner et al., 2007). Phosphorylation sites within the proteins of dragline silk (MaSp1 and MaSp2) were identified by mass spectrometry (dos Santos-Pinto et al., 2014; dos Santos-Pinto et al., 2015; Santos-Pinto et al., 2016) (Table 4). Protein glycosylation is responsible for the adhesive qualities of aggregate glue. It is estimated that more than 80% of threonine residues in aggregate proteins are *O*-glycosylated (Tillinghast et al., 1992). In the first three subgroups of the AgSp1 protein high serine/threonine regions are seen which are likely glycosylated (Stellwagen and Renberg, 2019). Aggregate proteins in *N. clavipes* are reported as glycosylated proteins. Multiple *O*-glycosylation and one possible *N*-glycosylation site were predicted in these proteins (Choresh et al., 2009). Moreover, Flag proteins present PTMs such as 45 hydroxylated proline residues as well as phosphorylation and nitrotyrosination sites (Table 4). Since these hydroxylation residues are located in the GPGGX motifs, this may explain the mechanoelastic property of these fibres (dos Santos-Pinto et al., 2018). These PTMs may cause changes in protein conformation and thus influence the properties of the proteins and interactions with other proteins as well as storage and self-assembly of silk proteins (Heim et al., 2009; Santos-Pinto et al., 2016). There is still no information about the recognition motifs and glycosylation patterns on spider silk proteins. Due to the lack of knowledge on spidroin PTMs, the PTM system of a production host might not fit the needs of the spider protein molecules (Peng et al., 2020). Future studies of spider silk proteomes can provide more details concerning PTMs of spidroins contributing towards a better understanding of their effects on mechanical properties, fibre assembly and solubility as well as selection of appropriate expression hosts.

**TABLE 4:** Detected/predicted PTMs on spider silk proteins.

Silk/protein	Species	Detected/predicted PTMs				Detection method	References
		Phosphorylation	Hydroxylation	*O*-glycosylation	*N*-glycosylation		
Dragline	*N. clavipes*			Glycosylation was confirmed. However, the pattern of glycosylation and the type of spidroin (MaSp1 or MaSp2) were not identified		Concanavalin A	Sponner et al. (2007)
Spidroin1	*N. clavipes*	The major PTMs in spidroin-1	yes	N.D.	N.D.	Gel-based MS strategy involving CID and ETD fragmentation	dos Santos-Pinto et al. (2014)
	*N. edulis*	8 sites on S, Y					
	*N. madagascariensis*	2 sites on S, Y					
		4 sites on S, Y					

Spidroin2	*N. clavipes*	36 sites on S, Y, T		N.D.	N.D.	Gel-based MS strategy involving CID and ETD fragmentation	Santos-Pinto et al. (2016)
Aggregate	*A. aurantia*			Yes		Gas-LC	Tillinghast et al. (1992)
Aggregate	*N. clavipes*			Yes	Yes		Choresh et al. (2009)
Aggregate	*A. trifasciata*			Yes	Yes		Stellwagen and Renberg (2019)
Flag	*N. clavipes*	on Y	45 hydroxylated P residues	N.D.	N.D.	NanoLC-ESI-CID/ETD-MS	dos Santos-Pinto et al. (2018)
			The recognition motif is GPGGS				

PTMs, post translational modifications; S, serine; Y, tyrosine; T, threonine; P, proline; LC, liquid chromatography; ESI, electrospray ionisation; CID, collision-induced dissociation; ETD, electron-transfer dissociation; MS, mass spectrometry; N.D., no data.

### Limitations of Spider Silk Production

Milligram amounts of natural dragline silks can be harvested by forcibly milking spiders, or by collecting the egg sacs to retrieve tubuliform silks. However, this process is expensive and time-consuming. For example, one million *N. madagascariensis* spiders and more than 70 working individuals are needed to make 3.4 m textile from natural dragline silk, with an estimated cost of over $500,000 (Vierra et al., 2011). Moreover, the cannibalistic nature of most spiders make them unsuitable for livestock breeding. Consequently, recombinant production of spider silk spidroins attracted interest in research. Different production hosts such as bacteria, yeasts, insects, mammalian cells, animals and plants have been used to produce recombinant spider silk proteins. Since these proteins are very long with a multitude of highly repetitive sequences, and therefore difficult to express in full length, currently the main strategy is to design and produce chimeric spidroins. Recombinant spidroins after production are spun into fibres through different methods (reviewed by Belbeoch et al., 2021; Koeppel and Holland, 2017). Besides other challenges, which we discuss below, a rigid down-stream processing is necessary when the silks are intended to be used as implantable biomaterial (Decker, 2018).

#### Bacterial Systems

*Escherichia coli* is one of the most used systems for recombinant protein production. *E. coli* cells have rapid growth, high productivity, potential for scale-up production and low production cost (Chen, 2012). Consequently, bacteria were used to produce recombinant spider silk proteins (Bhattacharyya et al., 2021). However, low expression level was the most prominent issue which might be due to inefficient transcription and translation. This problem was attributed to the repetitive core domain of sequence resulting in the high demand for glycyl-tRNA. The lack of tRNA or codon choices may cause premature translation termination (Fahnestock and Irwin, 1997b; Xia et al., 2010). To overcome this issue, a metabolically engineerd *E. coli,* in which the glycyl-tRNA pool was elavated, could express the 284.9 kDa protein of *N. clavipes.* A repeat motif 34 bp from MaSp1 partial cds of *N. clavipes* (accession number M37137) (Table 5) was used in this research. The yield was 1.2 g/L after purification, and the tenacity, elongation and Young’s modulus were similar to those of *N. clavipes* dragline silk fibres (Xia et al., 2010).

**TABLE 5:** The lengths of complete cds of spidroins genes with their accession numbers in the NCBI.

Accession number	Spider species	Silk type	Gene	Intron	Length of cds (bp or kb)
M37137	*Nephila clavipes*	Dragline	*MaSp1*	No	Partial cds, 2247 bp
EF595246	*Latrodectus Hesperus*	Dragline	*MaSp1*	No	Complete cds, Single exon, 9390 bp
KF032719.1	*Cyrtophora moluccensis*	Dragline	*MaSp1s*	No	Complete cds, 1,320 bp
EF595245	*Latrodectus hesperus*	Dragline	*MaSp2*	No	Complete cds, Single exon, 11340 bp
JX112872	*Argiope bruennichi*	Dragline	*MaSp2*	No	Complete cds, 10083 bp
U47855.1	*Araneus diadematus*	Dragline	*ADF3*	No	Partial cds, 1911 bp
U47856.1	*Araneus diadematus*	Dragline	*ADF4*	No	Partial cds, 1233 bp
JX513956	*Araneus ventricosus*	Minor ampullate	*MI*	Yes, 5628 bp	Complete cds, 5440 bp (after removal of the single intron)
AF027972 AF027973	*Nephila clavipes*	Flag	*Flag*	Yes	Partial cds
AH009146					
KY398016.1	*Argiope argentana*	Pyriform	*PySp1*	No	Complete cds, 17277 bp
MH376748	*Araneus ventricosus*	Pyriform	*PySp1*	No	Full length, 11931 bp
MN704282	*Araneus ventricosus*	Pyriform	*PySp2*	No	Complete cds, 6468 bp
MG021196	*Araneus ventricosus*	Aciniform	AcSp1	No	Complete cds, 10338 bp
MT078766	*Araneus ventricosus*	Aciniform	AcSp2	No	Complete cds, 14238 bp
MF192838	*Araneus ventricosus*	Tubuliform	*TuSp1*	No	Complete cds, 5763 bp
MK138561.1	*Argiope trifasciata*	Aggregate	*AgSp1*	Yes, 6690 bp	Gene sequence: ∼49 kb
					Complete cds: 42270
—	*Mastophora phrynosoma*	Aggregate	*AgSp2*	Yes, ∼ 37.5 kb	Gene sequence: over 100 kb
					Complete cds: ∼62 kb
EU780014.1	*Nephila clavipes*	Aggregate	*AGS1*	—	Complete cds, 1221 bp, 2145 bp
Eu780015.1			*AGS2*		

bp, base pair; kb, kilo base; cds, coding sequence.

To produce spider silk protein with longer size in *E. coli*, the split inteins mediated ligation technique was utilized. DNA part assembly allows for repeat motifs assembly by digestion and enzymatic ligation. A single repeat unit from MaSp1 was used to create up to 192-mer protein. Proteins with a molecular mass of 556 kDa were created at a yield of 2 g/L or 63 mg/g cell dry weight. Tensile strength and modulus were estimated from 1.03 ± 0.11 GPa to 13.7 ± 3.0 GPa, respectively. These recombinant proteins have similar mechanical properties as their natural counterparts. Fibre to fibre variation likely comes from genetic instability (Bowen et al., 2018) which affects final mechanical properties. These recombinant proteins do not possess N- and C-terminals, which may be the reason for shorter fibres. To dissolve spidroin powders, hexafluro-2-propanol (HFIP) was used which is a harsh reagent and is not a long-term sustainable approach (Zhang et al., 2019). Recombinant MaSp1s proteins with a yield of 300–400 mg/L of induced culture medium were produced in *E. coli* BL21 (Thamm and Scheibel, 2017). Forming inclusion bodies (IBs) is one of the problems in the production of recombinant spider silks in *E. coli*. Often, harsh conditions are used to solve this problem, for example dissolving proteins in a high concentration of urea or guanidine hydrochloride which often results in the poor recovery of bioactive proteins. In order to solve this problem, a mild solubilization strategy was used including a one-step heating method in the presence of low concentration of urea (Cai et al., 2020). Extensive purification is another limitation of using this host. Ammonium sulfate precipitation is inconvenient and time-consuming, and nickel columns are too expensive for large scale production and purification.

Sustainable cell factory platforms are being developed due to the awareness of climate change, food and water crises and depletion of fossil resources. These platforms should be able to employ sustainable bioprocesses and depend solely on renewable non-food bioresources as feedstocks. For the first time, an MaSp1 protein was produced using photosynthetic and halophilic bacteria under sea water conditions. A purple nonsulfur bacterium, *Rhodovulum sulfidophilum*, has been developed as a potential alternative platform to replace heterotrophic microbial cells. Biological contamination risk could be decreased due to the capacity to grow under seawater. Some challenges still need to be resolved, for example the demand for glycine and alanine tRNAs, and genetic stability of constructs (Foong et al., 2020).

The combination of motifs of different types of spider silk proteins was reported recently. To expand mechanical properties of recombinat spider silk fibres, two libraries of genes from *L. hesperus* were created. Library A and B consists of *masp1, masp2, tusp1, acsp1* and *acsp1, pysp1, misp1, flag* respectively*.* Random ligation of different spidroin genes from library A or B resulted in new proteins with new mechanical properties. Higher elastic moduli were seen in the samples from library A compared to those of library B. In comparison with natural silk proteins, both libraries had higher elastic moduli (Jaleel et al., 2020). In other research, a chimeric protein of Flag-AcSp1 was expressed in *E. coli* with a molecular mass of 36.8 kDa. The mean diameter of fibres was estimated to be 1–2 µm. These fibres have a toughness of ∼33.1 MJ/m^3^ and a tensile strength of ∼261.4 MPa (Tian et al., 2020) (Supplementary Table S1). However, all bacterial systems lack the capacity for proper PTMs on the recombinant proteins and thus may limit their use.

#### Yeast, Insect Cell Line and *Bombyx mori* Silkworm

*Pichia pastoris* is another organism that has been used to express dragline silk spidroins and considered as a proper replacement to *E. coli* when it comes to efficient production. Additionally, this yeast can secrete the recombinant proteins (Heidebrecht and Scheibel, 2013). The first report on expressing spidroin1 in *Pichia pastoris* GS115 is related to Fahnestock and Bedzyk (1997b) (Supplementary Table S1). This host cell was also used to produce the 2E12 protein, a 113.6 kDa analogue of MaSp2 from *Nephila madagascariensis* (Bogush et al., 2011)*.* Earlier, successful production of 1F9, an analogue of MaSp1, which encodes a 94 kDa protein in *Saccharomyces cerevisiae* was demonstrated and the tensile strength of 0.1–0.15 GPa and elasticity of 5–15% were measured (Bogush et al., 2009)*.* The production level was 450 mg/L in *Saccharomyces cerevisiae* (Sidoruk et al., 2015). Some challenges are identified with using *P. pastoris* such as poor expression in shake flasks and the need for bioreactors, proteolysis and self-assembly *in vivo* (Werten et al., 2019).

Insect cells have also been taken into consideration for research studies of spider silk proteins production. The most important reason is that the evolutionary distance between spiders and insects is relatively small. Cell line Sf9, derived from the fall armyworm *Spodoptera frugiperda*, was employed to express two dragline proteins of *Araneus diadematus,* ADF3 and ADF4, targeted to the cytosol. Spidroin proteins of 60 kDa were reported in the cytosol with 5 mg/L of insect cell culture. This research showed coiled filaments forming within the cytoplasm. These filaments ranged in diameter from 200 nm to 1µm, and the length was up to 100 µm. Since the length of filaments was too short due to cell size limitation, mechanical force measurement failed (Huemmerich et al., 2004). Moreover, complicated cloning steps and time-consuming regeneration were drawbacks (Heidebrecht and Scheibel, 2013).

Silkworms are good candidates for producing recombinant spider silk as they are able to spin spider silks into fibres due to their natural spinning apparatus. With the TALEN (transcription activator-like effector nucleases) strategy, the silkworm fibroin heavy chain gene was substituted with the *MaSp1* gene (1.6 kb), and transformed cocoon shells contained up to 35.2% MaSp1 protein (Xu et al., 2018). In addition, with CRISPR/Cas9 technology, spider silk proteins of native size were successfully produced. The *MaSp1* gene (6 kb) was incorporated into the genome of *Bombyx mori.* The silkworm fibroin heavy or light chain (FibH or FibL) intron (*FibH*) was replaced with the spider silk gene. For insertion, the intron region was used to ensure that any CRISPR/Cas9-induced sequence changes have no influence on protein production. FibH or FibL-spider silk fibres generated mechanical properties like natural silk (1.2 GPa). The transgenes were stable through subsequent generations. This study shows the feasibility of silkworms as a natural spinner for industrial production (Zhang et al., 2019).

#### Mammalian Cell Lines and Transgenic Animals

Mammalian cells are alternative expression systems for the production of recombinant proteins. Successful expression of dragline silk proteins of *N. clavipes* (MaSp1 and MaSp2) and *A. diadematus* (ADF3) was reported (Lazaris et al., 2002) in two different cell lines: bovine mammary epithelial cells excelling at secreting proteins outside the cell, and hamster kidney cells adapting to produce large amounts of recombinant protein. Both cell-lines secreted soluble proteins. The recombinant production yield ranged from 25 to 50 mg/L. The data for toughness, modulus and strain break was estimated as 0.64–0.85 gpd (gram per denier), 42.8–110.6 gpd and 43.4–59.6% (Lazaris et al., 2002). In a further attempt, Nexia Biotechnologies tried to produce these proteins in goat milk (Vendrely and Scheibel, 2007). These goats produced MaSp1 and MaSp2 protein analogues with approximately 65 kDa (Karatzas et al., 2007; Copeland et al., 2015), but the quantity was very low (Vendrely and Scheibel, 2007) (Supplementary Table S1). The silk protein purification process from transgenic goat milk is long, expensive and inefficient. To increase production, the TFF (Tangential Flow Filtration) process was optimized, and spider silk proteins were recovered at approximately 0.5 g/L (Yazzie et al., 2017). To increase the purity and quantity of recombinant spider silk proteins, the CRISPR/Cas9 system was used. The alpha-s2-casein gene coding for the native milk protein in goat was replaced with the MaSp1 gene (2046 bp). The average maximum stress was 21–73 MPa (Decker, 2018).

Mice are other transgenic animals that produced MaSp1 and MaSp2 proteins with a molecular mass of 40 kDa in milk, however, the tensile strength was lower than that of natural silk (Xu et al., 2007). Recently, the development of transgenic sheep embryos was reported. Using a liposome-mediated method, sheep fibroblasts were transfected with plasmids containing a spidroin gene. The aim of this study was to produce recombinant spidroins in hair follicles of sheep. The authors could successfully develop the method, and pregnancy was observed. However, no offspring was produced (Li et al., 2020).

#### Plant Systems

In tobacco leaves, recombinant MaSp1 and MaSp2 proteins were produced with a molecular mass of 60.3 and 58.5 kDa. Both proteins were targeted to the ER by means of PR1b (secretory signal peptide from tobacco) and accumulated in this organelle due to the ER retention signal KDEL. The data from Western blotting showed the single bands for proteins in stable expression, however, a protein ladder appeared for the transient expression of MaSp1. Some assumptions such as premature termination, unstable rearrangement of the T-DNA, endogenous plant proteases, trypsin-like cleavage sites and protease activity during leaf wounding were presented as an explanation. Moreover, different codon usage would be another reason for this protein ladder. The maximum yield of 0.0025 and 0.025% total soluble protein (TSP) were estimated for MaSp1 and MaSp2 proteins, respectively. Two promoters were used to determine their impact on protein yields. Transformed tobacco with tCUP (tobacco cryptic constitutive promoter) produce less amounts of MaSp1/MaSp2 than those plants with CaMV-35S (cauliflower mosaic virus) promoter (Menassa et al., 2004), and there is no data for the mechanical properties of extracted proteins (Supplementary Table S1).

In other research, the DPIB-8p proteins (synthetic analogue of spidroin1 with a molecular mass of 64 kDa) were targeted to the apoplast, ER lumen and vacuole by fusing the sporamin signal peptide, sporamin propeptide, and KDEL peptide to investigate the organelle potential and to enhance the accumulation of recombinant proteins. The accumulation level in the apoplast and ER of *Arabidopsis* leaves was 8.5 and 6.7% TSP, but retention in the vacuole faced failure. In addition, spidroin proteins could accumulate in ER and vacuole of *Arabidopsis* seeds to 18 and 8.2% TSP (Yang et al., 2005). Previously, recombinant spidroins (64 and 127 kDa) were produced in seeds and leaves of *Arabidopsis* and soy somatic embryos wihout taking protein targeting approaches. The synthesis of 64 kDa DB1B monomers showed equal molecular size, while minor by-products also occurred during the 127 kDa DP1B synthesis. The yield in these plants ranged from 0.03 to 1.2% TSP (Barr et al., 2004).

In order to produce larger recombinant proteins, the intein-based multimerization technique was utilized. This resulted in the production of Flag spidroin multimers longer than 250 kDa in the ER of tobacco leaves. The yield was estimated at 1.8 mg/50 g leaf material. In terms of physical properties, fibrillae with a diameter ranging from approximately 1–2 µm and a length of up to 500 µm were detectable (Hauptmann et al., 2013a). However, mechanical analyses such as tensile strength and toughness were not investigated. Post-translational multimerization *in vitro* is a further way to produce spider silk multimers with a large size. Transglutamination is a crosslinking of specific aa motifs allowing for more or less multimerization of proteins. The K-MaSp1-100xELP (K: lysine-tagged, ELP: elastin-like polypeptide) and Q-MaSp1-100xELP (Q: glutamine-tagged) cassettes were engineered. After purification of monomers Q-MaSp1-100xELP and K-MaSp1-100xELP, recombinant microbial transglutaminase (rMTG) treatment *in vitro* was performed to cross-link the monomers. This multimerization resulted in fusion proteins of more than 250 kDa. Atomic force microscopy (AFM) measured the elastic penetration modulus of the samples. This value measures the stiffness of a solid material (E value, GPa). Layers of recombinant spider silk fusion monomers, such as multimers linked via Q-/K-tags, were produced by casting (Weichert et al., 2014). Indeed, the highest E value (3.29 ± 0.03) was measured for Q-/K-MaSp1-100xELP cross-linked multimers compared to monomers of Q- or K-tagged MaSp1. Thus, the stiffness increased with multimerization (Weichert et al., 2014).

The same designed *Flag* gene was used to study the dimerization of a Flag monomer via cysteines in the C-terminus. The C-terminal domain of MaSp and Flag contain one or two cysteines, which are thought to crosslink the spidroins during assembly via disulfide bridges. Nevertheless, the results raised many questions. Half of the Flag monomers were dimerized. It was uncertain whether dimerization by the C-terminal domain affects fibre formation (Hauptmann et al., 2013b). To date, it has not been tested again in plant hosts.

The advantages of long-term storage recombinant spider silk in tobacco seeds were studied. Here, protein multimers larger than 450 kDa were synthesised by the intein-based multimerization technique. The GPGGX and GGX motifs of Flag partial cds (accession numbers AF027972 and AF027973), *LeB4* legumin signal peptide and ER retention signal KDEL were used in the construct. The intein-mediated self-splicing and ligation of proteins did not occur straight after translation. Hence, high molecular Flag proteins appeared only after a while. The study showed no decrease in the accumulation or loss of multimerization of spider silk proteins in seeds over 8 weeks at 15°C with 49% humidity. Also, long-term storage and expression of the synthetic Flag in seeds over two or three generations were stable (Weichert et al., 2016). In comparison to bacterial cells, the yield of 190 mg/kg obtained for USP-FIC (unknown seed protein promoter-flag intein c-myc) expressed in plants was low (Whittall et al., 2020). Distinct multimeric bands were visible due to the protein multimerization process with different sizes. Variable sizes of linear multimers, multimerization of epitope tag as well as the lack of N and C-terminal areas of Flag spidroin within the construct can be described as limitations of that technique.

One of the important problems of spider silk protein production in plant systems so far are low yields. For more efficient purification, ELP (elastin-like polypeptide) repeats were used to produce recombinant spider silk proteins in tobacco and *Solanum tuberosum*. ELP consists of Val-Pro-Gly-Xaa-Gly (Xaa is any amino acid except proline). The purification method for ELP is named ITC (heat denaturing and Inverse Transition Cycling) which is simple, scalable and inexpensive. ELPs are water-soluble below a specific temperature and turn into insoluble proteins when the temperature rises. Oligomeric repeats of ELP were fused to the spider silk sequence and after expression and purification, up to 400 mg spider silk proteins could be isolated from 6 kg of tobacco leaves. This report is the highest amount of spider silk proteins purified from plants (Scheller et al., 2004; Hauptmann et al., 2013b; Heppner et al., 2016).

Synthetic genes of 2000–6000 bp based on the sequence of *MaSp2* were expressed in *Medicago sativa* (alfalfa) leaves and resulted in proteins of 80–110 kDa. The yield was not determined due to storage problems. The synthetic spidroins produced in alfalfa did not freeze well, so the proteins were insoluble, making extraction and purification impossible. Thus, pure recombinant spidroins and measurable yields could not be achieved (Hugie, 2019).

Methods for the production of synthetic spider silk-like proteins in corn endosperm or plant shoot tissue were provided (Sylvester et al., 2015). In rice, recombinant spidroins of 22 kDa were produced and could successfully reduce blood glucose levels in diabetic mice. No data was reported for the production yield in transgenic rice (Park et al., 2019) (Supplementary Table S1). To date, except for MaSp1/MaSp2 and Flag, no other spider silk protein has been recombinantly produced in plant systems.

### Alternative Production Systems

The global demand for recombinant proteins has lead to research on transgenic microalgae as production hosts. Some features such as rapid growth, stable transgenic transformation, cost-effective production, scalable production as well as the ability to produce complex proteins with PTMs are promising. Attempts were made to produce a chimeric protein consisting of an antimicrobial protein from a bacteriophage and a spider silk protein in *Chlamydomonas reinhardtii,* which has a GC-rich genome, and thus may be well suited to produce spider silk proteins. The rationale was that recombinant spider silk proteins may act as a support for other proteins. A proposed application for these studies is the development of artificial skin for burn victims. However, the low yield of recombinant proteins (0.2% TSP) produced in microalgae is a big challenge and hinders commercial scale production. Several strategies including codon optimization, development of vectors and using proper promoter and terminator were presented to increase the expression level (Specht et al., 2010; Rasala and Mayfield, 2011).

**FIGURE 3:**
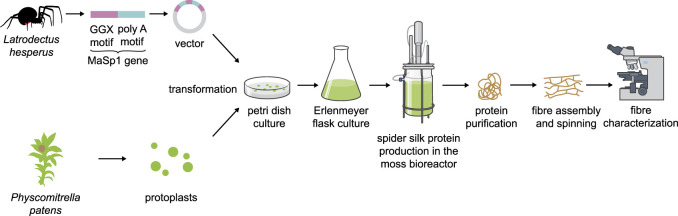
Schematic overview of spider silk protein production in Physcomitrella.

Another alternative production platform could be the moss Physcomitrella. Mosses are used in a wide variety of biotech applications, from carbon capture in peatlands to cosmetics (Decker and Reski, 2020). Physcomitrella especially has a proven track record in molecular farming with several candidate biopharmaceuticals being produced in this host (Reski et al., 2015), in which glyco-engineering of PTMs is possible due to precise and efficient genome engineering (Decker et al., 2014). Transformation of protoplasts, subculture and production in simple inorganic media devoid of sugars, growth factors and antibiotics in Petri dishes, Erlenmeyer flasks and photobioreactors are well established for this platform (Reski et al., 2018; Figure 3). Surprisingly, even human complement factor H (FH) can be produced in Physcomitrella (Büttner-Mainik et al., 2011) and is fully active in pre-clinical trials (Michelfelder et al., 2017). This difficult-to-express protein has a molecular mass of 155 kDa, several repeat units and intra-molecular disulfide bonds, and is heavily glycosylated. In contrast to other plant systems, Physcomitrella accepts a variety of animal sequences for protein production (Gitzinger et al., 2009) and addition of additives or co-expression of supporting proteins can stabilize the secreted protein product (Baur et al., 2005). Moreover, a detailed analysis of the Physcomitrella genome and of expression patterns for human cDNAs led to a codon-optimization tool that resulted in drastically enhanced protein yield and purity for FH and for human blood-clotting factor IX (Top et al., 2021), also making Physcomitrella a promising candidate for the production of spider silk proteins.

Large amounts of recombinant spidroins are required for spinning the fabrics, and the host system able to produce spidroins on a large scale in the bioreactor is a logical choice (Whittall et al., 2020). A recombinant spidroin containing only the repetitive domain might have problems with solubility or exhibit premature fold (Peng et al., 2016). However, this problem could be circumvented by either adding native N- and C-terminals as flanking regions to improve solubility or by fusing ELPs and targeted spidroin for selective precipitation (Heidebrecht and Scheibel, 2013; Peng et al., 2016).

## Conclusion and Outlook

**FIGURE 4:**
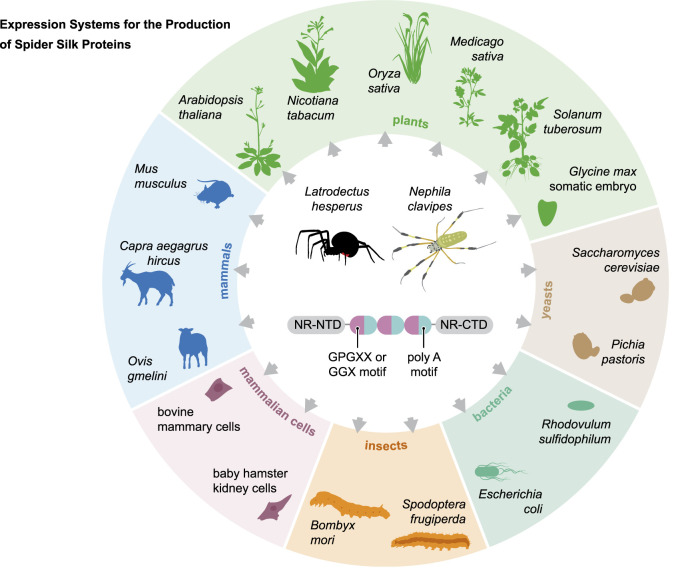
Overview of the expression systems used in the production of recombinant spider silk protein.

Spider silks have attracted interest for many years due to their superior properties in combination with biodegradability and biocompatibility. As reviewed in this paper, many expression systems (Figure 4) have been used to develop suitable production systems for the efficient production of recombinant spider silk proteins. The long length and highly repetitive nature of spider silk genes make these attempts challenging. To overcome these problems, chimeric genes have been used to produce chimeric spidroins. With extensive metabolic engineering, the production of large spider silk proteins could be possible in a bacterial system. The solubility of spidroins produced in *E. coli* remains challenging (Whittall et al., 2020). Some spidroins such as dragline and aggregate are glycosylated proteins, however, bacterial systems are not capable to produce such PTMs.

Plants are already in use for the production of enzymes, carbohydrates, lipids (Ray and Behera, 2017), biodegradable plastic-like compounds (Moire et al., 2003) and other proteins such as collagen (Haagdorens et al., 2021). Plant molecular farming can be integrated with material research, for example, to produce next-generation vaccines. Instead of using polymeric materials as nanocarriers, recombinant-expressed VNPs (virus nanoparticles) can be used which are very stable and can withstand temperatures outside cold chain requirements (Chung et al., 2021). Plants offer several advantages over conventional eukaryotic and prokaryotic expression platforms. In comparison to mammalian cell cultures, plants are safe and the risk of contamination with human pathogens is low (Hauptmann et al., 2013b; Buyel, 2019). In terms of cost of goods, plant systems are generally more competitive (Dove, 2002). The ability to produce correctly folded complex and posttranslationally modified proteins is another benefit of plant-based expression systems compared to bacterial systems (Hauptmann et al., 2013b). However, plant expression systems for spider silk production have faced a challenge (Chung et al., 2012). Recombinant spidroins have yielded from micrograms to about 200 mg per kilogram of plant tissue, which is still less than the commercially acceptable level (1–5 g/kg). New production hosts and strategies such as gene optimization, metabolic engineering and purification methods may allow spider silk protein production on an industrial scale.

## Data Availability

The original contributions presented in the study are included in the article/Supplementary Material, further inquiries can be directed to the corresponding author.
