# Supplementary material for: Recombinant Spider Silk: Promises and Bottlenecks
Source: Front Bioeng Biotechnol. 2022 Mar 8;10:835637. doi: 10.3389/fbioe.2022.835637 (PMC8957953; doi:10.3389/fbioe.2022.835637)
Supplement: Supplementary file 1 [file Table1.DOCX]

Supplementary Table 1. Recombinant production of spider silk proteins in different organisms. MS: moleculare mass, RP: recombinant protein, kDa: kilodalton, YM: Young’s modulus, MPa: megapascal, GPa: gigapascal, TSP: total soluble protein, mg: milligram, L: litre, ER: endoplasmic reticulum, TD: terminal domain, Ni-NTA: nickel nitrilotriacetic acid, HisPrep FF: histidine prepacked fast flow, HiPrep SP: histidine prepacked small protein, SSP: sporamin signal peptide, SP: sporamin propeptide, FPLS: fast protein liquid chromatography, His-tag: histidine tag, tCUP: tobacco cryptic constitutive promoter, USP-FIC: unknown seed protein promoter-flag intein c-myc, ITC: heat denaturing and inverse transition cycling, ELP: elastin-like polypeptide, LeB4: seed specific legumin B4 protein, PR1b: pathogenesis-related 1b protein, N.D.: no data.

| **Organism** | **Spider species** | **Protein** | **Method** | **Target compartment- Signal peptide** | **MS of RP (kDa)** | **Purification method** | **Yield** | **Mechanical properties** | **Reference** |
| --- | --- | --- | --- | --- | --- | --- | --- | --- | --- |
| **Bacteria** | | | | | | | | | |
| *E. coli* BL21(DE3) | *N. clavipes* | MaSpI |  |  | 284.9 | Acidic precipitation followed by a fractional ammonium sulfate precipitation. | 1.2 g/L | Tenacity:  508 ± 108 MPa  Elongation:15 ± 5%  YM:21 ± 4 GPa | Xia et al., 2010 |
| *E. coli*  NEB 10-beta (NEB10β) | *N. clavipes* | MaSpI | Split inteins-based multimerization |  | 556 | Acidic precipitation followed by a fractional ammonium sulfate precipitation. | 2 g/L or 63 mg/g cell dry weight. | Tensile strength:  525-1031 MPa  YM:7.8-13.7 GPa | Bowen et al., 2018 |
| *E. coli* BL21(DE3) | *L. hesperus* TD,  *C. moluccensis* core domain | MaSpIs |  |  | 42 | Ni-NTA sepharose column | 300-400 mg/L of induced culture medium | Strength:282 ± 66 MPa  Toughness:  144 ± 44 MJ/m^3^  YM:1.5 ± 0.3 GPa | Thamm and Scheibel, 2017 |
| *R. sulfidophilum* |  | MaSp1 |  |  | ~31 | HisPrep™ FF 16/ 10 20 mL column | 3-10 mg/L | N.D | Foong et al., 2020 |
| *E. coli* | *L. hesperus* | Mixture of different spidroins |  |  | 11.5-81.4 | Ni-NTA beads | 50-100 mg/L | Both libraries had higher elastic moduli rather than natural silk proteins | Jaleel et al., 2020 |
| *E. coli* | *A. ventricosus*  *A. trifasciata* | Flag-AcSp1 |  |  | 36.8 | Ni-NTA columns | 50 mg/L | Toughness:~33.1 MJ/m^3^  Tensile strength:  ~261.4 MPa | Tian et al., 2020 |
| **Yeast** | | | | | | | | | |
| *P. pastoris* GS115 (*his4*) | *N. clavipes* | MaSp1 |  |  | 65 |  | 663 mg/L | N.D. | Fahnestock and Bedzyk, 1997 |
| *P. pastoris* | *N. madagascariensis* | 2E12 |  |  | 113 | HiPrep SP column in the FPLS system. |  | N.D. | Bogush et al., 2011 |
| **Organism** | **Spider species** | **Protein** | **Method** | **Target compartment- Signal peptide** | **MS of RP (kDa)** | **Purification method** | **Yield** | **Mechanical properties** | **Reference** |
| *S. cerevisiae* | *N. clavipes* | MaSp1 (1F9) |  |  | 94 |  | 450 mg/L | Tnsile strength:  0.1-0.15 GPa  Elasticity:5-15 % | Bogush et al., 2009; Sidoruk et al., 2015 |
| **Insect** | | | | | | | | | |
| Cell line Sf9, derived from  *S. frugiperda* | *A. diadematus* | ADF-3  ADF-4 |  | Cytosol | 60 |  | 50 mg/L of insect cell culture | Diameters:200 nm-1µm  Lengths:up to 100 µm | Huemmerich et al., 2004 |
| *B. mori* silkworm | *N. clavipes* | MaSp1 |  |  | 67 |  | 35.2% of composite proteins | Breaking stress:  371.5 ± 27.5 MPa  Breaking energy:  84.8 ± 14.4 MJ/m^3^  YM:8.9 ± 1.3 GPa | Xu et al., 2018 |
| *B. mori* silkworm | *N. clavipes* | MaSp1 |  |  | 120-300 |  | N.D. |  | Zhang et al., 2019 |
| **Mamalian cells** | | | | | | | | | |
| Bovine mammary cells and baby hamster kidney (BHK) cells | *N. clavipes*  and *A. diadematus* | MaSpI, MaSpI (2)  MaSpII, ADF-3  ADF-3- His,  ADF-33, ADF-333 |  |  | 59,106,59, 60, 63, 110,  140 | His-tag was used for purification | 25 to 50 mg/L | Toughness (gpd):  0.645-0.895  Modulus (gpd):  42.8-110.6  Tenacity (gpd):1.8-2.26 | Lazaris et al., 2002 |
| **Transgenic animals** | | | | | | | | | |
| Goat |  | MaSp1 and MaSp2 |  |  | 65 |  | N.D. |  | Karatzas et al., 2007; Copeland et al., 2015 |
| Goat |  | MaSp1 |  |  | ~70 |  | N.D. | 21-73 MPa | Decker, 2018 |
| Mouse |  | MaSp1 and MaSp2 |  |  | 40 |  | 11.7 mg/L |  | Xu et al., 2007 |
| Sheep embryo |  |  |  |  |  |  | Offspring was not produced |  | Li et al., 2020 |
| **Plant** | | | | | | | | | |
| Tobacco | *N. clavipes* | MaSpI  MaSp2 |  | ER-PR1b and KDEL | 60.3  58.5 |  | 0.025% TSP in the 35S plants, <0.005% TSP in the tCUP plants. | N.D. | Menassa et al., 2004 |
| *Arabidopsis* leaves | *N. clavipes* | MaSpI  (DPIB) |  | Apoplast, ER, vacuole /  Combinations of SSP, SP, and KDEL | 64 |  | 8.5 % TSP  6.7 % TSP  None | N.D. | Yang et al., 2005 |
| **Organism** | **Spider species** | **Protein** | **Method** | **Target compartment- Signal peptide** | **MS of RP (kDa)** | **Purification method** | **Yield** | **Mechanical properties** | **Reference** |
| *Arabidopsis* seeds | *N. clavipes* | MaSpI  (DPIB) |  | Apoplast, ER, vacuole/  Combinations of SSP, SP, and KDEL | 64 |  | None  18 % TSP  8.2 % TSP | N.D. | Yang et al., 2005 |
| *Arabidopsis* leaves  *Arabidopsis* seeds  Soybean somatic embryos | *N. clavipes* | MaSpI (DPIB)  MaSpI (DPIB)  MaSpI (DPIB) |  | Without protein-targeting approach | 64, 127    64, 127    64, 127 |  | 0.34% and 0.03%  1.2% and 0.78%  1% and None  (%TSP) | N.D. | Barr et al., 2004 |
| Tobacco and potato leaves | *N. clavipes* | SO1  (SO1-100$\times$ELP) |  | ER | 94.2 | Heating, acidification and salt fractiation | 80 mg /kg of tobacco leaves | N.D. | Scheller et al., 2004 |
| Tobacco leaves | *N. clavipes* | FLAG | Intein-based multimerization | ER /  LeB4 and KDEL | > 250 | Based on Ni-NTA agarose | 1.8 mg/50 g leaf material. | Length:500 µm  Diameters:1-2 μm | Hauptmann et al., 2013 |
| Tobacco leaves | *N. clavipes* | MaSp1 | Transglutaminat-ion | ER | > 250 | ITC | Up to 400 mg from 6 kg of tobacco leaves | YM E:3.29±0.03 GPa | Weichert et al., 2014; Heppner et al., 2016 |
| Tobacco seed | *N. clavipes* | FLAG | Intein-based multimerization | ER /  LeB4 and KDEL | > 460 | Semi-quantitative analysis | 20-190 mg/kg for USP-FIC lines |  | Weichert et al., 2016 |
| Alfalfa  (*M. sativa*) | *N. clavipes* | MaSp2 |  |  | 80 |  | N.D. |  | Hugie, 2019 |
| Rice  (*Oryza sativa*) | *A. ventricosus* | AvMaSp |  |  | 22 |  | N.D. |  | Park et al., 2019 |

Barr, L. A., Fahnestock, S. R., and Yang, J. (2004). Production and purification of recombinant DP1B silk-like protein in plants. *Mol. Breed.* 13, 345–356. doi:10.1023/B:MOLB.0000034089.92263.ff.

Bogush, V. G., Sidoruk, K. V., Davydova, L. I., Zalunin, I. A., Kozlov, D. G., Moisenovich, M. M., et al. (2011). Recombinant analogue of spidroin 2 for biomedical materials. *Dokl. Biochem. Biophys.* 441, 276–279. doi:10.1134/S1607672911060093.

Bogush, V. G., Sokolova, O. S., Davydova, L. I., Klinov, D. V., Sidoruk, K. V., Esipova, N. G., et al. (2009). A novel model system for design of biomaterials based on recombinant analogs of spider silk proteins. *J. Neuroimmune Pharmacol.* 4, 17–27. doi:10.1007/s11481-008-9129-z.

Bowen, C. H., Dai, B., Sargent, C. J., Bai, W., Ladiwala, P., Feng, H., et al. (2018). Recombinant Spidroins Fully Replicate Primary Mechanical Properties of Natural Spider Silk. *Biomacromolecules* 19, 3853–3860. doi:10.1021/acs.biomac.8b00980.

Copeland, C. G., Bell, B. E., Christensen, C. D., and Lewis, R. V. (2015). Development of a Process for the Spinning of Synthetic Spider Silk. *ACS Biomater. Sci. Eng.* 1, 577–584. doi:10.1021/acsbiomaterials.5b00092.

Decker, R. E. (2018). Production and biocompatibility of spider silk proteins in goat milk. PhD Dissertation. Utah State University

Fahnestock, S. R., and Bedzyk, L. A. (1997). Production of synthetic spider dragline silk protein in *Pichia pastoris*. *Appl. Microbiol. Biotechnol.* 47, 33–39. doi:10.1007/s002530050884.

Foong, C. P., Higuchi-Takeuchi, M., Malay, A. D., Oktaviani, N. A., Thagun, C., and Numata, K. (2020). A marine photosynthetic microbial cell factory as a platform for spider silk production. *Commun. Biol.* 3, 1–8. doi:10.1038/s42003-020-1099-6.

Hauptmann, V., Weichert, N., Menzel, M., Knoch, D., Paege, N., Scheller, J., et al. (2013). Native-sized spider silk proteins synthesized in planta via intein-based multimerization. *Transgenic Res.* 22, 369–377. doi:10.1007/s11248-012-9655-6.

Heppner, R., Weichert, N., Schierhorn, A., Conrad, U., and Pietzsch, M. (2016). Low-tech, pilot scale purification of a recombinant spider silk protein analog from tobacco leaves. *Int. J. Mol. Sci.* 17, 1687. doi:10.3390/ijms17101687.

Huemmerich, D., Scheibel, T., Vollrath, F., Cohen, S., Gat, U., and Ittah, S. (2004). Novel Assembly Properties of Recombinant Spider Dragline Silk Proteins. *Curr. Biol.* 14, 2070–2074. doi:10.1016/j.cub.2004.11.005.

Hugie, M. R. (2019). Expression systems for synthetic spider silk protein production. PhD Dissertation. Utah State University.

Jaleel, Z., Zhou, S., Martín-Moldes, Z., Baugh, L. M., Yeh, J., Dinjaski, N., et al. (2020). Expanding canonical spider silk properties through a DNA combinatorial approach. *Materials (Basel).* 13, 3596. doi:10.3390/MA13163596.

Karatzas, costas n, Turner, jeffrey d, and Lazaris-Karatzas, A. (2007). Production of Biofilaments in Transgenic Animals. US. Patent No 7,157,615. 2. Available at: https://patents.google.com/patent/US7157615B2/en.

Lazaris, A., Arcidiacono, S., Huang, Y., Zhou, J. F., Duguay, F., Chretien, N., et al. (2002). Spider silk fibers spun from soluble recombinant silk produced in mammalian cells. *Science.* 295, 472–476. doi:10.1126/science.1065780.

Li, H., Chen, S., Piao, S., An, T., and Wang, C. (2020). Production of artificial synthetic spidroin gene 4S-transgenic cloned sheep embryos using somatic cell nuclear transfer. *Anim. Biotechnol.*, 1–11. doi:10.1080/10495398.2020.1737098.

Menassa, R., Zhu, H., Karatzas, C. N., Lazaris, A., Richman, A., and Brandle, J. (2004). Spider dragline silk proteins in transgenic tobacco leaves: Accumulation and field production. *Plant Biotechnol. J.* 2, 431–438. doi:10.1111/j.1467-7652.2004.00087.x.

Park, J.-E., Jeong, Y. J., Park, J. B., Kim, H. Y., Yoo, Y. H., Lee, K. S., et al. (2019). Dietary exposure to transgenic rice expressing the spider silk protein fibroin reduces blood glucose levels in diabetic mice: The potential role of insulin receptor substrate-1 phosphorylation in adipocytes. *Dev. Reprod.* 23, 223–229. doi:10.12717/dr.2019.23.3.223.

Scheller, J., Henggeler, D., Viviani, A., and Conrad, U. (2004). Purification of spider silk-elastin from transgenic plants and application for human chondrocyte proliferation. *Transgenic Res.* 13, 51–57. doi:10.1023/B:TRAG.0000017175.78809.7a.

Sidoruk, K. V., Davydova, L. I., Kozlov, D. G., Gubaidullin, D. G., Glazunov, A. V., Bogush, V. G., et al. (2015). Fermentation optimization of a *Saccharomyces cerevisiae* strain producing 1F9 recombinant spidroin. *Appl. Biochem. Microbiol.* 51, 766–773. doi:10.1134/S0003683815070066.

Thamm, C., and Scheibel, T. (2017). Recombinant production, characterization, and fiber spinning of an engineered short major ampullate spidroin (MaSp1s). *Biomacromolecules* 18, 1365–1372. doi:10.1021/acs.biomac.7b00090.

Tian, L. Y., Meng, Q., and Lin, Y. (2020). Expression and characterization of chimeric spidroins from flagelliform-aciniform repetitive modules. *Biopolymers* 111, 1–8. doi:10.1002/bip.23404.

Weichert, N., Hauptmann, V., Helmold, C., and Conrad, U. (2016). Seed-specific expression of spider silk protein multimers causes long-term stability. *Front. Plant Sci.* 7, 1–9. doi:10.3389/fpls.2016.00006.

Weichert, N., Hauptmann, V., Menzel, M., Schallau, K., Gunkel, P., Hertel, T. C., et al. (2014). Transglutamination allows production and characterization of native-sized ELPylated spider silk proteins from transgenic plants. *Plant Biotechnol. J.* 12, 265–275. doi:10.1111/pbi.12135.

Xia, X. X., Qian, Z. G., Ki, C. S., Park, Y. H., Kaplan, D. L., and Lee, S. Y. (2010). Native-sized recombinant spider silk protein produced in metabolically engineered *Escherichia coli* results in a strong fiber. *Proc. Natl. Acad. Sci. U. S. A.* 107, 14059–14063. doi:10.1073/pnas.1003366107.

Xu, H.-T., Fan, B.-L., Yu, S.-Y., Huang, Y.-H., Zhao, Z.-H., Lian, Z.-X., et al. (2007). Construct synthetic gene encoding artificial spider dragline silk protein and its expression in milk of transgenic mice. *Anim. Biotechnol.* 18, 1–12. doi:10.1080/10495390601091024.

Xu, J., Dong, Q., Yu, Y., Niu, B., Ji, D., Li, M., et al. (2018). Mass spider silk production through targeted gene replacement in *Bombyx mori*. *Proc. Natl. Acad. Sci. U. S. A.* 115, 8757–8762. doi:10.1073/pnas.1806805115.

Yang, J., Barr, L. A., Fahnestock, S. R., and Liu, Z. Bin (2005). High yield recombinant silk-like protein production in transgenic plants through protein targeting. *Transgenic Res.* 14, 313–324. doi:10.1007/s11248-005-0272-5.

Zhang, X., Xia, L., Day, B. A., Harris, T. I., Oliveira, P., Knittel, C., et al. (2019). CRISPR/Cas9 initiated transgenic silkworms as a natural spinner of spider silk. *Biomacromolecules* 20, 2252–2264. doi:10.1021/acs.biomac.9b00193.
